# Bridging the Gap: Using Consensus to Explore Entrustment Decisions and Feedback Receptivity in Competency-Based Emergency Medicine Residency Programs Through the Construction of a Q-Sample Incorporating a Delphi Technique

**DOI:** 10.3389/fmed.2022.879271

**Published:** 2022-06-02

**Authors:** Yu-Che Chang, Renee S. Chuang, Cheng-Ting Hsiao, Madalitso Khwepeya, Nothando S. Nkambule

**Affiliations:** ^1^Chang Gung Medical Education Research Centre (CG-MERC), Chang Gung Memorial Hospital, Taoyuan, Taiwan; ^2^Department of Emergency Medicine, Chang Gung Memorial Hospital, Taoyuan, Taiwan; ^3^College of Medicine, Chang Gung University, Taoyuan, Taiwan; ^4^Department of Emergency Medicine, Chang Gung Memorial Hospital, Chiayi, Taiwan; ^5^International Graduate Program of Education and Human Development (IGPEHD), College of Social Sciences, National Sun Yat-sen University, Kaohsiung, Taiwan

**Keywords:** competency-based medical education (CBME), Delphi technique, emergency medicine, Q-methodology, Q-sample, feedback receptivity, entrustment decisions, medical education

## Abstract

**Background:**

Recent changes in medical education calls for a shift toward student-centered learning. Therefore, it is imperative that clinical educators transparently assess the work-readiness of their medical residents through entrustment-based supervision decisions toward independent practice. Similarly, it is critical that medical residents are vocal about the quality of supervision and feedback they receive. This study aimed to explore the factors that influence entrustment-based supervision decisions and feedback receptivity by establishing a general consensus among Taiwanese clinical educators and medical residents regarding entrustment decisions and feedback uptake, respectively.

**Methods:**

In Q-methodology studies, a set of opinion statement (i.e., the Q-sample) is generated to represent the phenomenon of interest. To explore the factors that influence entrustment-based supervision decisions and feedback receptivity, a Q-sample was developed using a four-step approach: (1) literature search using electronic databases, such as PubMed and Google Scholar, and interviews with emergency clinical educators and medical residents to generate opinion statements, (2) thematic analysis and grouping using The Model of Trust, the Ready, Wiling, and Able model, and the theory of self-regulated learning, (3) translation, and (4) application of a Delphi technique, including two expert panels comprised of clinical educators and medical residents, to establish a consensus of the statements and validation for a subsequent Q-study.

**Results:**

A total of 585 and 1,039 statements from the literature search and interviews were extracted to populate the sample of statements (i.e., the concourse) regarding entrustment-based supervision decisions for clinical educators and feedback receptivity emergency medicine residents, respectively. Two expert panels were invited to participate in a Delphi Technique, comprised of 11 clinical educators and 13 medical residents. After two-rounds of a Delphi technique, the panel of clinical educators agreed on 54 statements on factors that influence entrustment-based supervision decisions and were categorized into five themes defined by the Model of Trust. Similarly, a total of 60 statements on the factors that influence feedback receptivity were retained by the panel of medical residents and were categorized into five themes defined by the Ready, Willing, and Able model and the theory of self-regulated learning.

**Conclusion:**

Though not exhaustive, the key factors agreed upon by clinical educators and medical residents reflect the characteristics of entrustment-based supervision decisions and feedback receptivity across specialties. This study provides insight on an often overlooked issue of the paths to teaching and learning in competency-based residency training programs. Additionally, incorporation of the Delphi technique further adds to the existing literature and puts emphasis as an important tool that can be used in medical education to rigorously validate Q-statements and develop Q-samples in various specialties.

## Introduction

A fundamental aspect of competency-based residency training is the decision to entrust a medical resident with the responsibility to care for a patient with minimal supervision (1). When made negligently, the decision can compromise patient safety. Clinical educators (CEs) rely on multiple sources of information to provide them with knowledge regarding how much supervision a medical resident may need during clinical training (2, 3). Entrustment decisions regarding supervision requires not only for CEs to reflect on their decision making process, but also for medical residents to reflect on their own skillset and characteristics (4, 5). Evidence suggest that the multidimensional nature of entrustment decisions are largely subjective and are both task dependent and task independent (3, 6–8).

To date, only a few studies have used a Delphi technique to build a consensus regarding how experienced CEs make entrustment-based supervision decisions in clinical practice (9, 10). Although these studies have established a consensus and shared mindset on factors that influence entrustment decisions among CEs (9, 10), they solely focus on the aspects related to a medical residents' characteristics without including that of CEs. Literature has shown, however, that these decisions can be influenced by the nature of the task and a supervisors' individual characteristics (6). Therefore, further research is needed to establish a consensus on the path to entrustment while taking into consideration the nature of the task and a CEs individual attributes (11). To contribute to the generalizability of factors considered to be important when making entrustment decisions, research in various countries should be conducted (9, 12). Results from such research can be used to establish standard of practice in regards to supervision decisions made by CEs in the clinical workplace (13).

Supervision facilitates learning in the clinical workplace and requires guided interactions between CEs, medical residents, and patients, giving medical residents exposure to hands-on practice (14, 15). The process of supervision allows CEs to provide a foundation for medical residents as they engage in direct practice (15) while the process of feedback empowers medical residents with the necessary knowledge to improve their clinical and procedural skills (15, 16). Feedback in the clinical setting manifests in many forms, it can be either explicit—given after assessment or focused on overall performance (i.e., written and verbal), or implicit (i.e., body responses and reactions) (17). Research has shown that entrustment decisions made after evaluating a medical resident's behaviors are, therefore, a reflection of their displayed level of competency (18). As such, the level of autonomy assigned to medical residents based on entrustment decisions is perceived as a form of implicit feedback regarding their entrustable practices (17–19). A medical resident's perception of the level of autonomy given to them during clinical practice can affect how they view their own clinical competency and influence how self-evaluative judgment skills regarding their clinical practice is developed (18–20).

However, formative feedback alone does not always yield the expected impact on learners (21). Studies note a gap between feedback received and how it is implemented, highlighting the role recipients need to play as active synthesizers and evaluators of feedback in order for learning to take place (16, 22). Several factors have been noted to influence the credibility and reliability of feedback received, e.g., the delivery method (15, 16, 23). Previous research has emphasized the development of feedback delivery skills among CEs (16, 22). Though, recent studies suggest a shift in research to focus on incorporating a learner's perspective of feedback credibility and reliability is necessary to bridge the gap between feedback and its intended outcome (24, 25). Through these findings, we propose that a learner's evaluation of feedback is likely to bring new perspectives through establishing a consensus on the factors that influence feedback receptivity. Insight on how feedback is received by medical residents can be used to enhance CEs feedback delivery skills (23, 24).

This study is part of a larger multi-year study regarding the incorporation of competency-based medical education and its subsequent assessment methods in emergency medicine in Taiwan. Recently, Taiwan was named as an international hub for Accreditation Council for Graduate Medical Education regional faculty development (26). We consider the path to entrustment decisions and feedback receptivity as two separate but crossing paths. Therefore, this study aims to establish a consensus on factors that affect a CEs path to making entrustment-based supervision decisions and the factors that affect a medical residents' receptivity to feedback received. A concourse of statements regarding such topics of interest was gathered from literature and semi-structured interviews. Additionally, we used a Delphi technique to establish expert consensus on critical factors that influence both entrustment decisions among CEs and medical residents' feedback receptivity.

## Methods

### Setting and Participants

We conducted this study in Taiwan from March 2020 to June 2021. Emergency medicine was among the first specialties to implement competency-based residency training programs amid the recent medical education reform in Taiwan (27, 28). In competency-based residency training programs, all senior physicians are expected to take part in the training of residents and medical residents are required to actively engage in their own training by seeking feedback and reflecting on their own progress (27, 29). Therefore, participants of our study included clinical educators and medical residents of various specialties practicing medicine in Taiwan. Participants were recruited via snowball sampling, with initial contact made by the principal investigator to the participants.

### Q-Methodology and a Delphi Technique

Increasing in popularity, Q-methodology is a mixed-methods approach to exploring human subjectivity (30–32). The goal of a Q-methodology study is to reveal different patterns of thoughts and perspectives of participants through ranking and sorting statements on a continuum of meaningfulness (31, 33). It draws on both qualitative and quantitative methodologies for data collection and analysis. However, there remain concerns about the best practice for Q-sample development, limited guidance on constructing a Q-sample, and methods to measure the validity and reliability of a Q-sample (33, 34).

The Delphi technique is a group facilitative method used with the intention of developing an expert-based judgment on a chosen topic to reach group consensus involving panel members who are experts in a selected field. Panelists remain anonymous from one another to reduce dominant personalities influencing the consensus process by one or more experts (35, 36). While there is no consensus for the exact methodology for conducting a Delphi study in literature, it can be argued that a typical Delphi technique is comprised of five steps: (1) a problem is presented to a panel of experts, (2) panel members individually respond through a structured questionnaire or interview, (3) data is gathered, analyzed, and reworked toward collective agreement, (4) repetition of steps two and three for several rounds, as needed, and (5) collective agreement is achieved through statistical analysis (37). After each round of the Delphi technique, feedback is provided by the panel members. Thereafter, panel members are given the opportunity to discuss with the research team and change their opinions in subsequent rounds. The entire iterative process occurs until a consensus is reached.

### Constructing the Q-Sample for the Delphi Technique

**Figure 1:**
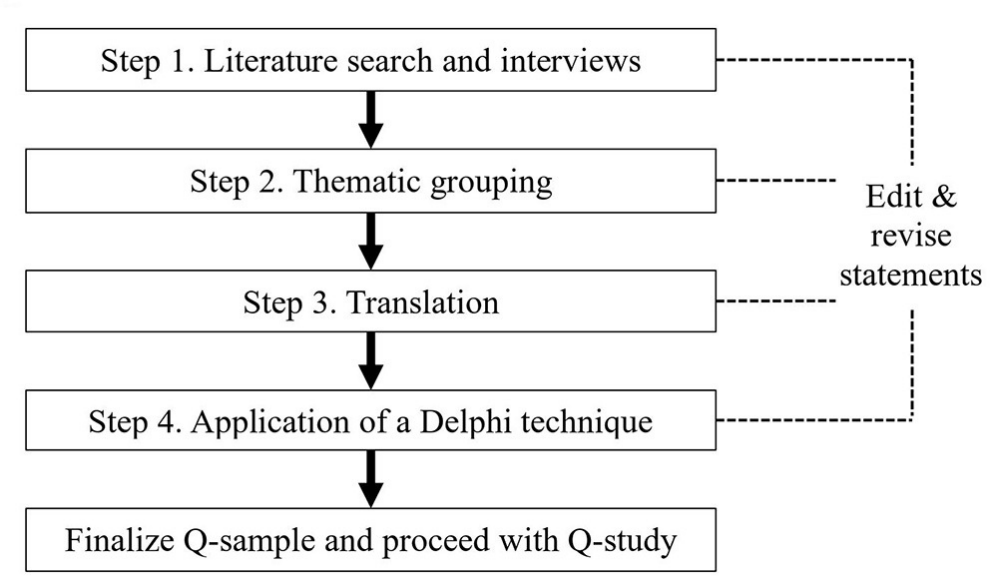
Four-step method for Q-sample construction.

Similar to the framework set by Kirschbaum et al. (38), construction of the Q-samples consisted of four steps and four points of refinement (Figure 1).

#### Step 1: Gathering Initial Thoughts—Literature Search and Interviews

Defining the concourse is the first step of a Q-methodology study. The concourse is a collection of possible statements that represent opinions of a given topic (39, 40). Using both ready-made (i.e., statements gathered from print media such as newspaper articles, magazines, and scientific literature) and naturalistic concourses (i.e., statements gathered from participants through questionnaires, interviews, and focus groups) (39, 41), opinion statements were gathered through a literature review and semi-structured interviews. Incorporating the use of scientific literature brought forth broader, cross-specialty perspectives regarding issues of entrustment decisions, clinical supervision, and feedback receptivity. Additionally, it facilitated the use of a Delphi technique for consensus by engaging CEs and medical residents from various specialties. Simultaneously, interviews with emergency CEs and EMRs was crucial to obtain specialty-specific viewpoints about clinical teaching, supervision, and learning through feedback in the emergency department and further guided the literature search. Material for the concourses aim to represent and capture the depth of existing opinions on the topic at hand-entrustment decisions and feedback receptivity (33, 40, 42).

##### Scientific Literature

A systematic search using PubMed and Google Scholar was independently conducted by three authors (NSN, RSC, and MK) from inception to April 2021. We used the following search terms for CEs: (clinical educator OR medical teacher OR supervisor OR attending physician) AND (decisions OR views OR attitude OR perceptions) AND (entrustment OR supervision OR scaffolding OR independent practice OR autonomous practice OR progressive independence). Additionally, the following search terms for EMRs: (resident OR medical trainee OR post graduate medical student OR emergency medicine resident) AND (feedback OR feedback synthesis OR feedback evaluation OR feedback perception OR feedback appraisal) AND (credibility OR reliability OR acceptability OR receptivity). Back referencing was used to identify potential studies and relevant citations to be included in our analysis.

Articles published in English and at any time were retained for analysis. Literature retained were those that met the following criteria: research published with clear relevance to the subject of factors that influence entrustment decisions by supervisors on trainees, research on feedback in medical education or medical training, performed in any country, and research focusing on factors affecting feedback receptivity. Quotes, statements, and key phrases that represented an opinion of a CE or medical resident concerning supervision, entrustable professional activities, entrustment decisions, and interactions between supervisors and trainees in the clinical setting were extracted. Additional articles were identified from reference lists of included papers.

##### Semi-structured Interviews

Semi-structured, one-on-one interviews with 13 emergency CEs and 11 EMRs were conducted to extract opinion statements and to observe for recurring themes. Interviews were conducted virtually through Zoom, a video-telephony propriety software program, audio recorded, and transcribed verbatim. CEs were initially prompted to answer questions regarding difficulties faced when making supervision-based entrustment decisions (Appendix 1). On the other hand, EMRs were initially prompted with questions regarding their opinions on the assessment tools used in their residency training programs, including strengths, weaknesses, and the credibility of feedback received (Appendix 2). Participants were affiliated with 9 different hospitals, each with emergency medicine residency training programs approved by the Residency Review Committee through the Taiwan Ministry of Health and Welfare.

To be eligible, emergency CEs interviewed had at minimum Five years of clinical teaching experience and were program directors or in a leadership position, while EMRs were in their third or 4th year of residency training. Details of the participants are shown in Table 1. The eligibility criteria was established to ensure participants had experienced the transition from traditional workplace-based assessment to competency-based medical education evaluation. Participants provided written informed consent prior to the interview and verbal consent prior to the audio recording. The interviews lasted between 30 and 60 min. Among emergency CEs, the goal of the interviews was to explore factors influencing entrustment while among EMRs, the goal was to explore feedback receptivity and how these decisions influenced their professional development and educational process.

**Table 1:** Demographics of interview participants (*N* = 24).

	Emergency clinical educators^*^N = 13	Emergency medicine residents^*^N = 11
**Sex**		
Female	2 (15.4)	4 (36.4)
Male	11 (84.6)	7 (63.6)
**Age**		
30–39	3 (23.1)	11 (100)
40–49	8 (61.5)	–
50 +	2 (15.4)	–
**Residency year**		
R3	–	6 (54.5)
R4	–	5 (45.5)
**Years of teaching experience**		
≤ 5	2 (15.4)	–
6–10	2 (15.4)	–
>10	9 (69.2)	–

* *Data presented as number (%)*.

#### Step 2: Thematic Grouping and Theoretical Considerations

Once all search efforts were exhausted and interviews were completed, all extracted information were refined into cohesive phrases and then into opinion statements. Like statements and statements with similar language or meaning were combined and grouped categorically using theoretical frameworks for each target population. The theoretical frameworks were used as guides to ensure that all possible viewpoints were accurately represented.

##### Clinical Educators

**Figure 2:**
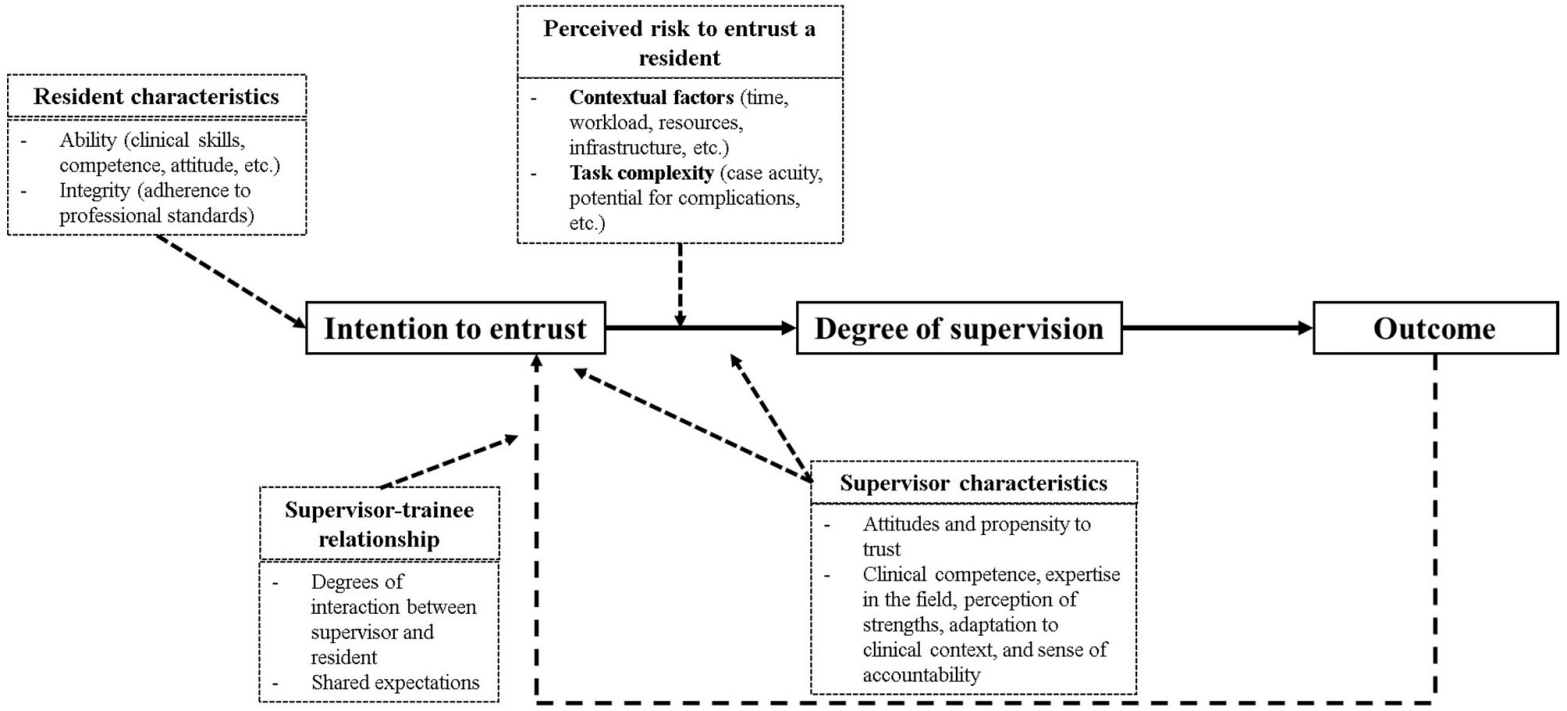
Model of trust adapted from the conceptual framework of the entrustment decision-making process (6).

Drawing from Roger Mayer and colleagues' Model of Trust (8), the conceptual framework of the entrustment decision making process aims to advance the understanding of how *ad-hoc* entrustment decisions are made in the day-to-day clinical practice (6) (Figure 2). The model illustrates the complexity of the entrustment decision making process in the clinical context, noting that these decisions are dependent on time, context, and the task at hand. Equally, these decisions are influenced by multiple factors related to the relationship fostered between CEs and medical residents (6, 7). Interactions among these factors determine the level of entrustment and supervision that a CE is likely to provide to a medical resident (6). Therefore, this model served as the foundation for thematic analysis of extracted statements derived from the aforementioned Step 1.

The extracted statements were categorized into five categories: (1) relationship between a supervisor and trainee, (2) contextual factors, (3) supervisor characteristics, (4) task complexity, and (5) trainee characteristics (6, 8). Every statement was assigned to a single thematic category.

##### Medical Residents

**Figure 3:**
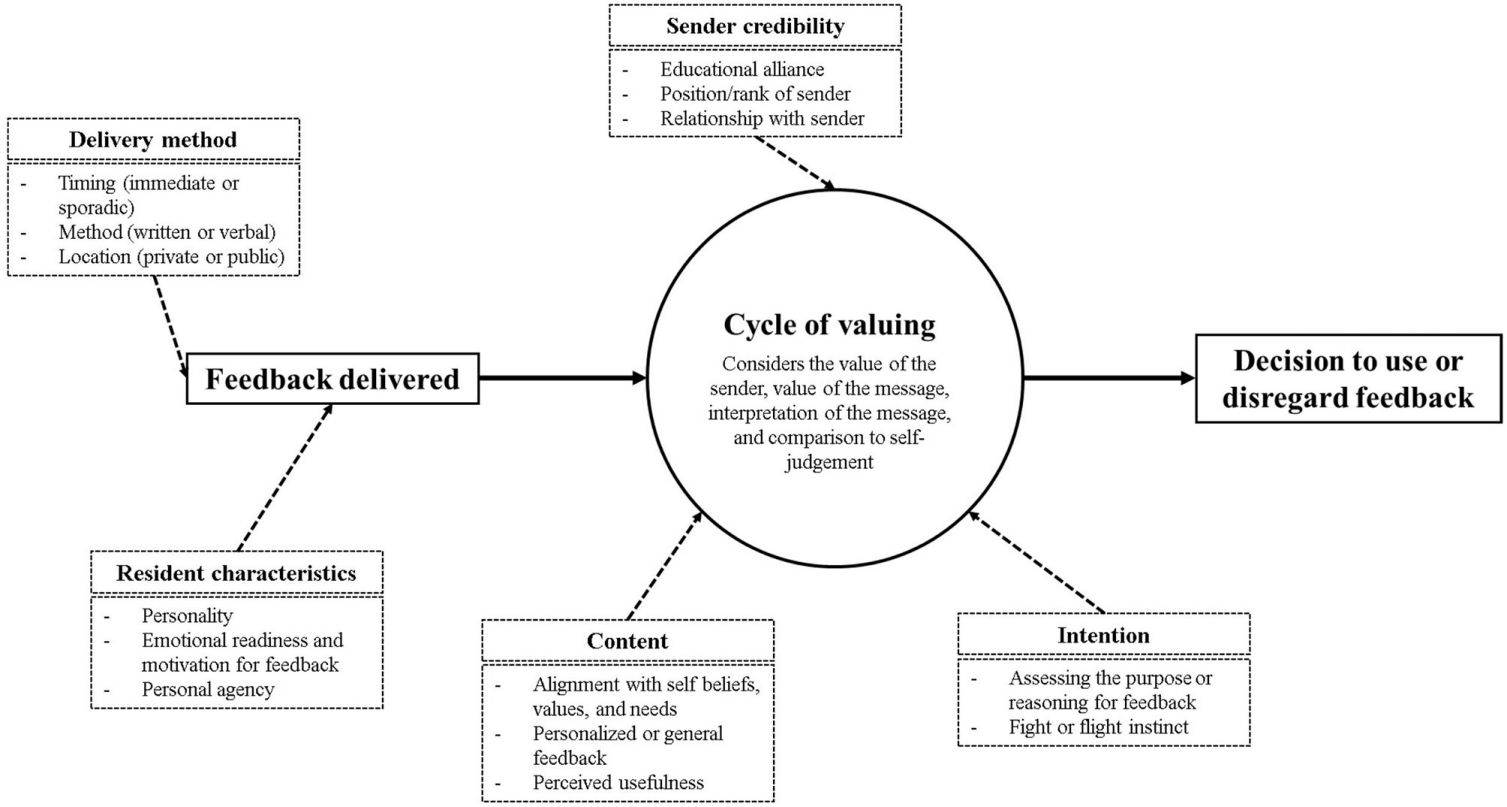
Cycle of feedback valuing derived from the five thematic categories derived from the Ready, Willing, and Able model and self-regulated learning (23, 43).

Feedback is essential in learning, however there are many factors that can influence the uptake of feedback by the learner. Developed by Garino (23), the Ready, Willing, and Able (RWA) model is a theoretical framework that explains the successful use of feedback (Figure 3). The model stipulates that upon receiving feedback, a learner goes through a cycle of valuing the messenger, valuing the message, makes meaning from the message, and compares it to their own self-evaluation. Once a learner has completed the cycle, they can then choose to engage in and employ adaptive learning strategies or dismiss the feedback received (23).

The successful use of feedback through the RWA model is set upon the foundation of self-regulated learning (SRL). SRL refers to the adaptive strategies that enables learners to transform their mental abilities into academic performance (23, 43). Applications of metacognitive strategies can be described through three phases of performance, reflection, and forethought (43, 44). Through the phases, learners receive feedback and align it to their personal goals, monitor their learning through self-questioning, and reflect on their learning to track improvements. The theoretical frameworks set by the theory of SRL and the RWA model demonstrates five categories for feedback receptivity, including: (1) delivery method, (2) characteristics of the learner, (3) intention and motivation to providing feedback, (4) content of the feedback, and (5) feedback sender credibility. Each statement was assigned to a single thematic category.

In the subsequent steps, statements extracted for emergency CEs and EMRs were revised for concise language while checking for simplicity, use of personal pronouns, and applicability to the cultural context. Negatively expressed statements were avoided because any disagreement with these statements may introduce a negative connotation into the proceedings and invoke a strong response from the participant (45). For example, if a statement were framed “I do not prefer written feedback,” a participant may disagree with this (i.e., give it a negative ranking) to negate the negative (*I do not*) and create the positive (*I do*) (45). Therefore, to avoid confusion or bias toward our statements, where a negative connotation was present, the statement was reworded to maintain a neutral (e.g., “written feedback”) or positive viewpoint (e.g., “I prefer written feedback”). Remaining statements were then proofread for grammar, reworded for clarity, and made certain each statement described only a single issue.

#### Step 3: Statement Translation

Statements were translated from English to Chinese to ensure target populations could fully understand the statement meanings. Forward translation from English to Chinese was done independently by the principal investigator, who is a native Chinese speaker. Then, back-translation was collaboratively done by two members of the research team, who are proficient in Chinese. Once back-translated, translated statements were compared to the original to ensure the intended conceptual meaning was maintained during the translation process. Any variances in the translations were reviewed and resolved within a consensus discussion among the research team. After discussion, repeated statements, statements with similar meanings, and those deemed irrelevant to the study were removed.

#### Step 4: Application of a Delphi Technique

A Delphi technique was employed to achieve expert consensus on pivotal factors that influence both entrustment-based supervision decisions and feedback receptivity. A multidisciplinary team of CEs and medical residents were recruited purposively to participate on the expert panel. Experts were defined as CEs and medical residents, practicing and training in various specialties. CEs had, at minimum 5 years of teaching experience, while medical residents were in their 3rd or 4th year of residency training to ensure that participants had experienced the recent change from assessment-based learning to competency-based training. Opinions on the number of statements in a Q-sample still varies among theorists and researchers. However, McKeown and colleagues suggest that a Q-sample of 50-70 is adequate for most studies (46). Through a discussion among the research team, a Q-sample of ≤60 was deemed ample to reduce the possibility of oversaturation or repetitive statements in this study. The Delphi technique was conducted from May 2021 to June 2021. All rounds of the Delphi technique were conducted via offline, paper surveys that were emailed as an attachment to participant individually.

Given that emergency medicine is a complex dynamic specialty that requires specific training in many disciplines (47), panel members from various specialties were invited to participate in the Delphi technique. As part of their residency training, EMRs will rotate between various specialties outside of emergency medicine (47). Therefore, it is crucial that the viewpoints of CEs from other specialties are included as they provide EMRs with feedback about their clinical competencies. Similarly, medical residents from various specialties may rotate in emergency medicine as part of their training. However, research has shown a discrepancy in reliability of feedback provided to non-EMRs who during emergency medicine rotations (48). Therefore it is imperative to understand the perspective of medical residents from various specialties as well on the comprehensibility and receptivity of feedback provided.

A panel of 11 CEs practicing in 10 specialties, including pediatrics (1), internal medicine (1), emergency medicine (2), orthopedics (1), dermatology (1), nephrology (1), nursing (1), critical care (1), thoracic surgery (1), and respiratory therapy (1) were included in the expert panel. Additionally, an expert panel of 13 medical residents training in 7 differing specialties, including obstetrics and gynecology (2), dentistry (2), emergency medicine (2), internal medicine (1), Chinese medicine (2), pediatrics (2), and general surgery (2) were invited.

##### Round 1

Through purposeful sampling, the expert panel was selected based on individual expertise and knowledge as follows: (1) current experience, and (2) from different specialties to facilitate diversity of views and experience in an effort to reduce both researcher and specialty bias.11 CEs and 13 residents from four hospitals were recruited for participation. Details of the panel members are shown in Table 2. First, an explanation of the study and its purpose was provided to each participant. CEs were instructed to rank their opinions of the statements regarding factors that affect entrustment decisions while supervising medical residents. On the other hand, medical residents were instructed to rank their opinions of the statements in regards to their receptivity of feedback that influence their learning. Sequenced according to the five thematic groups defined by the Model of Trust and SRL/RWA, respectively, the panel members were asked to rank the statements on a 5-point Likert scale ranging from 1 (unimportant) to 5 (most important). Additionally, panelists were asked to leave a comment on why they ranked the statement as such and to provide suggestions for improving statement clarity, if applicable. Once completed, the median and interquartile range (IQR) for the continuous variables were calculated as recommended by Jones and Hunter (49). An IQR of ≤ 1 for a 5-point Likert scale is suggested as an acceptable indicator of consensus (50–52). In this study, statements that reached expert consensus (i.e., median 5 and IQR ≤ 1) were deemed conclusive and were not reevaluated in subsequent rounds. Furthermore, to ensure that our final Q-samples were ≤ 60 statements, statements that achieved an IQR ≤ 1 and a median of 4 were reevaluated by panel members in Round 2.

**Table 2:** Demographics of panel members in the Delphi technique (*N* = 24).

	Clinical educators^*^^&^ *N* = 11	Medical residents^*^^&^ *N* = 13
**Sex**		
Female	4 (36.4)	8 (61.5)
Male	7 (63.6)	5 (38.5)
**Age**		
20-29	–	1 (7.7)
30–39	–	12 (92.3)
40–49	4 (36.4)	–
50–59	7 (63.6)	–
**Residency year**		
R1	–	2 (15.4)
R3	–	2 (15.4)
R4	–	3 (23.1)
R5	–	4 (30.8)
R6	–	2 (15.4)
**Years of teaching experience**		
≤19	6 (54.5)	–
>20	5 (45.5)	

* *Background of clinical educators invited from 4 different hospitals, including: pediatrics (1), internal medicine (1), emergency medicine (2), orthopedics (1), dermatology (1), nephrology (1), nursing (1), critical care (1), thoracic surgery (1), and respiratory therapy (1)*.

*#Background of medical residents in training invited from 2 different hospitals, including: obstetrics and gynecology (2), dentistry (2), emergency medicine (2), internal medicine (1), Chinese medicine (2), pediatrics (2), and general surgery (2)*.

& *Data presented as number (%)*.

##### Round 2

Participants from the first round of the Delphi technique were re-invited to participate in the second round to reevaluate statements that had reached consensus (i.e., IQR ≤ 1) but had a median of 4 to reduce the possibility of statement oversaturation. At the start, panelists were reminded of the study aim and explained the purpose of the second round (i.e., to reduce the number of statements and to obtain expert consensus). Median and IQR were once again calculated for each of the remaining statements to identify those that have reached expert consensus. Statements that reached expert consensus were revised as necessary based on feedback provided. Once Q-sample populations reached ≤ 60, no further rounds were necessary.

## Results

### Concourse Development and Statement Generation

**Figure 4:**
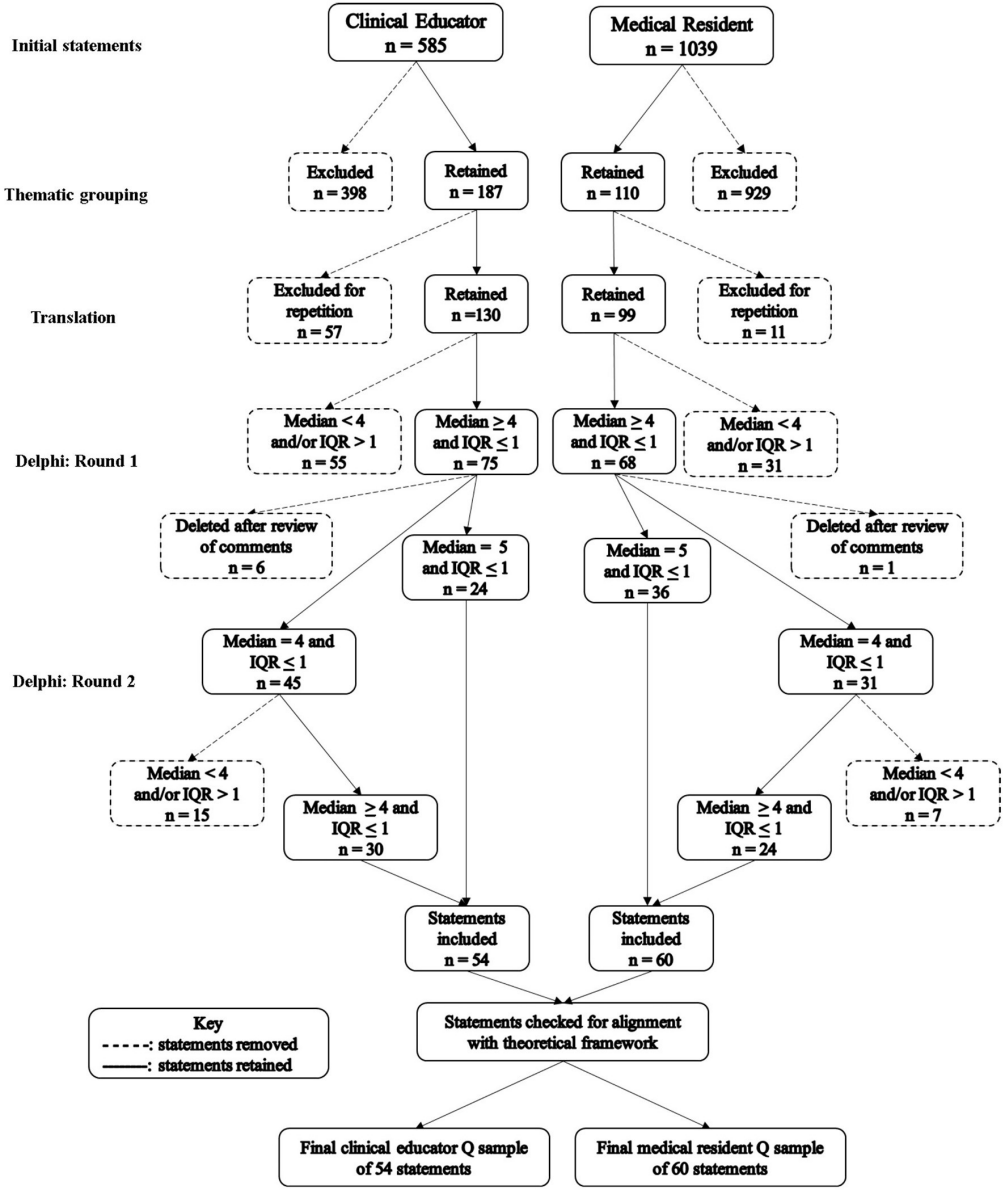
Results of statement selection and reduction.

In the process of identifying our concourse, a total of 585 and 1,039 statements from aforementioned interviews and scientific literature were extracted to populate the concourse for emergency CEs and EMRs, respectively, Figure 4 illustrates our concourse development and statement generation process. Of the 585 statements extracted for emergency CEs, 95 statements were derived from interviews with emergency CEs while 490 statements were derived from literature. After thematic sorting using the entrustment framework defined by the Model of Trust, 187 residual statements were retained. Similarly, of the 1,039 statements extracted for EMRs, 289 statements were gathered from interviews with EMRs and 750 statements were derived from literature. Then, 110 residual statements were combined and sorted thematically according to the five categories outlined by the SRL and RWA conceptual frameworks. Repeated statements and statements that did not reflect our research aims were removed. Upon translation of the statements, 130 statements and 99 statements were retained for CEs and EMRs, respectively.

### Building Consensus Through Delphi Technique

#### Round 1

From 130, a total of 75 statements achieved expert consensus among CEs. However, after a review of comments and suggestions from the 11 panelists, 6 statements were removed due to irrelevance or language ambiguity resulting in 69 statements retained. Similarly, of the 99 original statements, a total of 68 statements achieved expert consensus by the 13 medical residents However after evaluating comments from the panelists, one statement was deleted for ambiguity, leaving a total of 67 statements retained.

#### Round 2

A second round of a Delphi technique was employed to reevaluate important statements that did not achieve expert consensus among CEs and medical residents. Panel members from the first round of the Delphi technique were re-invited to reevaluate the remaining statements. The additional round of the Delphi allowed us to further reduce the number of statements and to achieve the goal of ≤60 statements.

##### Clinical Educators

Of the 75 statements from the first round, 69 statements achieved expert consensus, however 45 statements had reached median 4 and IQR ≤ 1 and were reevaluated in Round 2. Upon reevaluation, 30 statements achieved expert consensus. Therefore, after two rounds of a Delphi technique, panel members agreed on 54 statements (i.e., 24 statements from Round 1 and 30 statements from Round 2) on factors that influence entrustment-based supervision decisions (Table 3). Common factors mentioned in literature and interviews included their frequency of interaction with resident, a resident's level of training or seniority, and a resident's awareness of their skills accumulation and personal limitations to deliver safe patient care. Additionally, statements that were deemed context specific (i.e., statements that were repeatedly mentioned during interviews but infrequently found in literature) included a resident's ability to communicate with difficult family members, CEs sense of responsibility to educate a resident, and CEs attitude toward resident training responsibilities.

**Table 3:** Q-Sample statements (*N* = 54) mapped to the model of trust domains.

**Relationship between a supervisor and trainee (*N* = 2)**
1. Frequency of interaction with resident
2. Recent encounter with resident
**Contextual factors (*N* = 8)**
1. Workload during a shift
2. The number of trainees on duty under your supervision
3. Level of clinical case or task complexity
4. Patient's level of acuity
5. The clinical procedure's level of invasiveness
6. Resident's ability to handle complications when they arise
7. Level of risk associated with the clinical situation or task
8. The level of uncertainty of the case
**Supervisors characteristics (*N* = 8)**
1. Inclination to trust residents
2. Willingness to make decisions based on information provided by the resident
3. Willingness to take legal responsibility from the results of a resident's actions
4. Personal level of confidence as a clinical supervisor
5. Attending's level of competence or experience relating to the case
6. Personal medical education philosophy
7. Sense of responsibility to educate the resident
8. Attending's attitudes toward resident training responsibility
**Task complexity (*N* = 18)**
1. Residents' ability to set priorities for clinical tasks
2. Residents' ability to manage clinical task
3. Resident's accumulated experience in patient care
4. Resident's ability to adapt and change treatment plans according to changes in patient status
5. Resident's ability to handle clinical cases in a timely manner
6. Resident's ability to systematically perform clinical tasks
7. Resident's capacity for clinical reasoning
8. Resident's decision-making skills
9. Resident's expressed self-confidence to perform clinical tasks
10. Resident's ability to discuss their clinical reasoning with others
11. Resident's ability to think quickly in the case of adverse events or uncertain situations
12. Resident's interpersonal skills and ability to communicate effectively with the patient
13. Resident's ability to communicate with difficult patients or family members
14. Resident's ability to adapt appropriate communication style when discussing patient's conditions
15. Resident's attitude toward inter-professional colleagues
16. Resident's personality
17. Resident's level of empathy
18. Resident's tendency to make errors during patient care
**Resident characteristics (*N* = 18)**
1. Resident's overall displayed competence and medical knowledge
2. *ad-hoc* observation and evaluation of resident at work
3. Records of previous assessments
4. Resident willingness to take on challenging or unfamiliar tasks
5. Resident's willingness to seek help when encountering unexpected situations
6. Resident's awareness of their personal limits
7. Resident's ability to practice evidence-based medicine
8. Resident's level of professionalism
9. Resident's level of enthusiasm toward clinical work
10. Resident's level of engagement in their own professional development
11. Resident's sensitivity to treatment standards
12. Resident ability to remain unbiased when delivering patient care
13. Resident's ability to disclose significant information that can affect patient outcome
14. Resident's dependability in completing assigned tasks
15. Resident demonstrates honesty and humility
16. Resident's receptivity to guidance
17. Resident's receptivity to feedback
18. Resident's ability to use feedback to improve their clinical practice

All 54 statements were successfully assigned into one of the five categories defined by the Model of Trust: 2 statements into relationship between a supervisor and trainee, 8 statements into contextual factors, 8 statements into supervisor characteristics, 18 statements into task complexity, and 18 statements into resident characteristics. Furthermore, a resident's capacity for clinical reasoning, the complexity of the clinical case, and frequency of interaction with a resident were among the most mentioned factors in literature and in interviews that heavily influenced entrustment-based supervision decisions.

##### Medicine Residents

Among medical residents, 67 statements achieved expert consensus, however, 31 statements had reached median 4 and IQR ≤ 1 and were reevaluated in Round 2. Of these 31 statements, 24 statements achieved expert consensus. Therefore, a total of 60 statements (i.e., 36 statements from Round 1 to 24 from Round 2) on the factors that influence residents' receptivity to feedback were retained after Round 2 (Table 4). Factors that enhanced feedback receptivity ranged from feedback that is delivered in a clear and easy to understand manner to feedback from a source that has a good understanding of the curriculum. Furthermore, there were no statements deemed contextually specific among medical residents. All statements extrapolated from interviews were commonly found in international literature used in this study.

**Table 4:** Q-sample statements (*N* = 60) mapped to the SRL and RWA^*^ model domains.

**Delivery (*N* = 17)**
1. Face-to-face visual displays of feedback combined with written summaries
2. Feedback that uses common languages
3. Feedback that is clear and easy to understand
4. Feedback focusing on individual performance in a team setting
5. Feedback based on direct observation
6. Feedback that is communicated respectfully
7. Qualitative feedback addressing areas that need to be improved
8. Feedback provided in a safe and confidential environment
9. Feedback that can be monitored
10. Grade feedback combined with narrative feedback
11. Feedback immediately after completion of a specific skill or task
12. Feedback given in the middle of the training year
13. Feedback after clear standards for my performance have been communicated with me
14. Feedback delivered based on order of importance
15. Feedback at a critical time during the learning process
16. Feedback given when there is sufficient time to deliver it
17. Timely feedback given after direct observation and assessment
**Characteristics of the recipient (*N* = 4)**
1. Feedback that takes into consideration the resident's level of training and competence
2. Feedback that fosters interaction between clinical educator and resident
3. Feedback that does not cause the resident to fear or worry
4. Feedback that gives the resident confidence to seek more feedback
**Intention or motivation for providing feedback (*N* = 11)**
1. Feedback that motivates the resident to properly participate in learning activities
2. Feedback that points out there is room for residents to improve
3. Facilitative feedback that assists the resident in finding resources for learning
4. Constructive feedback that is supportive
5. Criticism delivered in a positive way to alleviate negative emotions
6. Feedback that is neutral
7. Feedback that is gender-neutral
8. Feedback that allows for future observation and follow-up
9. Feedback that fosters trust between clinical educator and resident
10. Feedback that motivates a resident to work toward a desired goal
11. Feedback that promotes self-evaluative judgment
**Content of the feedback (N = 13)**
1. Feedback that is based on a common goal between a clinical educator and resident
2. Feedback tailored toward personal professional development
3. Feedback that results in an action plan
4. Feedback about the quality of the performance on a specific task to track improvement
5. Feedback that points out errors in clinical judgment
6. Detailed feedback highlighting what went right, what went wrong, and what can be improved
7. Feedback tailored toward a specific task performed under direct observation
8. Feedback on how to improve in a specific area
9. Feedback about what the supervisor would do with a patient
10. Feedback from a holistic point-of-view that is both clinical and interpersonal skills
11. Feedback that focuses on general performance and attitude, not on any specific task
12. Feedback that details strengths and weaknesses with reasons
13. Feedback based on the professional work culture
**Sender credibility (*N* = 15)**
1. Feedback from an honest source
2. Feedback with a foundational basis
3. Feedback that portrays the sincerity of the supervisors
4. Feedback from patients on delivery of care
5. Multisource feedback for specific tasks
6. Feedback from a source that is willing to give criticism
7. Constructive feedback from a peer
8. Feedback from a reliable (trust—inherent quality of the evidence) source
9. Feedback from a credible (believable—whether or not you trust it) source
10. Feedback from a source that has a good understanding of the curriculum and is competent about the role
11. Feedback from a supervisor that has known me for a long time
12. Feedback from a supervisor I have worked with for a long time
13. Feedback including the supervisors sharing their experience
14. Feedback from a supervisor actively engaged in the learning process
15. Feedback from a respectable source

* *SRL, self-regulated learning; RWA, ready, willing, and able model*.

All 60 statements were successfully classified into the five categories defined by SRL and RWA: 17 statements into Delivery, 4 statements into characteristics of the recipient, 11 statements into intention or motivation for providing feedback, 13 statements into the content of the feedback, and 15 statement into Sender credibility. Furthermore, feedback provided in a safe and confidential environment, detailed feedback based on milestone assessments, and timely feedback provided after direct observation were among the most mentioned factors in literature and in interviews that influence feedback receptivity.

## Discussion

This present study aimed to understand factors that influence entrustment in supervision decisions and feedback receptivity by establishing a general consensus among CEs and medical residents, respectively. To our knowledge, this is the first study of its kind to explore these two paths within a competency-based learning context in emergency medicine through the simultaneous development of two Q-samples and a Delphi technique to obtain expert consensus. Through the use of consensus and the steps for constructing a Q-sample to explore entrustment-based supervision decisions and feedback receptivity, this study provided deeper insight into entrustment, supervision, and feedback receptivity in the clinical context.

### Entrustment Decisions and Clinical Supervision

This study revealed a sample of consensus-based factors that affect CEs decision to entrust medical residents with a clinical tasks. It aids in determining of the degree of supervision that is necessary to provide medical residents with the skills necessary to progress toward independent practice. Our study revealed factors that ranged from the frequency of interaction with a medical resident and their fostered relationship to factors regarding the context and level of risk associated with the clinical task. Globally, competency based medical education is rapidly emerging as the prominent paradigm across workplace-based education (53). Competency and milestone-based learning is designed to improve teaching and assessment of learners among broad domains (54). An important aspect of competency-based medical education is the decision to entrust a medical resident to perform clinical procedures, which is a complex task that does not come with a binary solution. Rather, it lies on a continuum from medical residents requiring a high degree of supervision and direct observation of procedures performed to medical residents receiving minimal supervision (55).

Though the decision to entrust a medical resident with a patient is a complex and subjective process, our study revealed the non-linear path toward deciding how CEs decide to reduce the levels of supervision and to entrust medical residents to perform patient care independently. By understanding key factors that CEs deem as key indicators of competency exhibited by an medical resident that influence their entrustment decisions, CEs and medical residents establish shared mental models that informs what constitute as competency in the clinical setting (56, 57). This shared mental model may guide new medical residents' on behaviors worth emulating and to continue behaviors worth reinforcing (56, 57). This can empower medical residents to participate in their learning process and contributes to efforts of instilling a student-centered learning culture in the clinical workplace (58).

Our factor statements also include statements related to task or situation dependent domains and a CEs characteristics. Insight into how various factors influence entrustment decisions is necessary for CEs reflective practice. Establishing consensus on a subjective matter, such as entrustment decisions, may reveal discrepancies between the theoretical assumptions and the reality of factors that influence their decisions in practice (59). It can reveal how a CEs personal and educational background along with contextual factors that form the hidden curriculum can influence supervision, teaching, and learning (60, 61). Previous studies have illustrated how a CEs first impressions of a medical resident tend to influence their subsequent entrustment and supervisions decisions, suggesting that training is essential to helping supervisors pay attention to this form of bias (62, 63). We suggest that by uncovering this shared mindset among CEs, our study can contribute to the discourse of how educators can modify their entrustment and supervision behavior to reduce bias.

### Feedback Comprehension and Receptivity

Under competency-based medical education, CEs are advised that learners receive timely, specific, constructive, and fair feedback to enhance their learning (64). The Delphi technique revealed factors that influence medical residents' synthesis of feedback and receptivity, ranging from the delivery method of the feedback to individual characteristics of the recipient and the credibility of the feedback deliverer. In line with findings from contemporary literature, our results describe the importance of using various feedback delivery methods, the content of the feedback, and the credibility of the feedback provider (23, 43). Therefore, these study results reflect an agreement among medical residents of factors that influence the comprehension, evaluation, and receptivity of feedback.

While feedback receptivity may be subjective in nature, our results reiterate factors that resonate most with medical residents across various specialties. Additionally, it bridges the gap between a theoretical understanding and the practical reality of what medical residents perceive useful feedback relative to other forms of feedback. Research has shown that without a common frame of reference and perceptions of quality feedback can be detrimental to a learner's engagement in their learning process and may prevent them from fully maximizing the benefits offered by formative feedback (65). Insights into medical residents' shared mindset on the relative importance of various factors that influence their receptivity to feedback is crucial in helping CEs develop the appropriate mechanism for giving feedback to residents.

### Study Implications

Our study serves as a foundation toward the development of initiatives designed to help CEs optimize their supervision practices through the learners' perspective as receivers of feedback through entrustment-based supervision decisions. We suggest that these decisions and practices should be personalized to the learner so that they reflect individual skillsets, which is essential in providing transparency in assessment. Feedback should be informed by CEs and that the level of entrustment and supervision constitutes as a form of feedback, aligning with previous studies that bridge supervision and feedback (66). This indicates that the amount of supervision assigned to trainees is a form of implicit feedback (17–19). Finally, this study contributes to the clinical supervision and feedback literature by providing representative factors that influence entrustment-based supervision decisions and feedback receptivity. This serves as a foundation for fostering a shared mental model between CEs and medical residents across various specialties as the cornerstones of entrustment-based supervision decisions and applicable feedback.

To our knowledge, this is the first study of its kind in constructing a concourse for a Q-methodology study through incorporating a Delphi technique to explore factors that influence supervision entrustment decisions among emergency CEs and EMRs feedback receptivity. Apart from using the two types of concourses (i.e., naturalistic and ready-made), the strength of this study lies at the use of three conceptual models to inform the thematic analysis of the statements. Using established theoretical models structures the construction of a Q-sample and ensures that statements represent different facets of the Research Topic (33, 38, 45). Although the statements extracted primarily originated from international literature, a Delphi technique was incorporated in our study to obtain validation from Taiwanese experts for relevance to the broader East Asian healthcare setting. Through using theoretical frameworks and a Delphi technique, the resulting Q-samples have greater plausibility for capturing a balance of rigor, depth, and comprehensiveness of the selected topics.

### Limitations

The development of a consensus among CEs and medical residents in this study was not without limitations. There was potential for researcher bias stemming from the subjective nature of the process of initial selection of statements and selection of the theoretical frameworks used. Though we used established conceptual models for entrustment decisions and feedback synthesis to structure the construction of our Q sample, the selected theories used to categorically organize our statements may not have been fully representative of all domains of supervision and feedback uptake. Therefore, the statements may not represent an exhaustive list of factors that influence entrustment decisions and feedback receptivity. While this study was limited to emergency CEs and EMRs in Taiwan, we invited a multidisciplinary panel of CEs and medical residents in an attempt to diversify opinions, as suggested by previous literature (38). At the time of writing there is no consensus in the literature defining the optimal panel size and composition when using a Delphi technique with literature, noting panel sizes ranged from 8 to 1,000 of panel members (38). This study invited a panel of 11 CEs and 13 medical residents given the reduced number of statements to maintain homogeneity and reduce oversaturation that could lead to unreliable data. Despite these limitations, the study presents a rigorous approach using strict criteria to establish consensus among CEs and medical residents across various specialties and to construct Q-samples to be used within the context of emergency medicine.

## Conclusion

Though not exhaustive, the key factors agreed upon by CEs and medical residents reflect characteristics of entrustment-based supervision decisions and feedback receptivity across specialties. Considering that the factors are consensus-based, they represent a two separate but complementary mindsets between CEs and medical residents. Our study provides insight on an often overlooked issue of the paths to teaching and learning in competency-based residency training programs. As such, our results can serve as a foundation to develop initiatives aimed at the professional development of CEs supervision skills and to enhance medical residents' participation in their own professional development. Additionally, this study aids in increasing transparency when making entrustment-based supervision decisions that can increase feedback receptivity. The incorporation of a Delphi technique further adds to the existing literature and places emphasis on an important tool that can be used in medical education research to rigorously validate Q-statements and develop Q-samples in various specialties.

## Data Availability Statement

The raw data supporting the conclusions of this article will be made available by the authors, without undue reservation.

## Ethics Statement

Ethical approval for this study was granted by the Chang Gung Medical Foundation Institutional Review Board (201900082B0).

## Author Contributions

Y-CC: conceptualization, methodology, formal analysis, resources, writing—original draft preparation, writing—review and editing, supervision, project administration, and funding acquisition. RC: methodology, formal analysis, writing—original draft preparation, and writing—review and editing. C-TH: formal analysis, data curation, writing—original draft, writing—review and editing, and project administration. MK: formal analysis, investigation, data curation, and writing—review and editing. NN: conceptualization, methodology, formal analysis, writing—review and editing, and supervision. All authors contributed to the article and approved the submitted version.

## Funding

The Ministry of Science and Technology is the Government Ministry of Taiwan for the promotion and funding of Academic Research, Development of Science and Technology and Science Parks (Funding Number: 108-2511-H-182-007-MY2).

## Conflict of Interest

The authors declare that the research was conducted in the absence of any commercial or financial relationships that could be construed as a potential conflict of interest.

## Publisher's Note

All claims expressed in this article are solely those of the authors and do not necessarily represent those of their affiliated organizations, or those of the publisher, the editors and the reviewers. Any product that may be evaluated in this article, or claim that may be made by its manufacturer, is not guaranteed or endorsed by the publisher.
