# Supplementary material for: Bridging the Gap: Using Consensus to Explore Entrustment Decisions and Feedback Receptivity in Competency-Based Emergency Medicine Residency Programs Through the Construction of a Q-Sample Incorporating a Delphi Technique
Source: Front Med (Lausanne). 2022 Jun 2;9:879271. doi: 10.3389/fmed.2022.879271 (PMC9201255; doi:10.3389/fmed.2022.879271)
Supplement: Supplementary file 1 [file Data_Sheet_1.docx]

**Appendix 1. Clinical educators' interview guide questions used to generate Q statement**

1. From a clinical teacher's role perspective, how difficult is it to entrust residents with clinical work?

- From the teacher and clinical teaching context stand point, do you think it is difficult to trust a resident?
- What are some of the difficulties of trusting residents?
- What do you think are the characteristics of a resident that can be entrusted? Or what do you think are the characteristics of a resident worthy of trust?
- What do you think are the characteristics of a resident that cannot be entrusted?
- When you work together [with a resident], how do you decide how much to trust him or her? What is that degree of trust based on?

2. Do you have an example that you can give to demonstrate the considerations you take into account when making decisions about how much trust you want to give to a resident?

3. What is your understanding of readiness for independent practice and level of supervision?

- How do you decide that the resident is ready to do a clinical task?
- How do you judge a resident's readiness for independent practice?
- How do you decide how much supervision to provide a resident? What kind of standard do you use, what kind of obstacles do you face

4. What do you think is the purpose of the educational assessment?

- Do you think the assessment method reflects the ability of students?

5. What role does evaluation/ assessment play in determining a resident's level of preparedness and the level of supervision you give to a resident?

- Do you think the results of the evaluation help you decide the level of supervision to give to a resident?
- How do you use assessment results to judge a resident's readiness for practice?
- How does assessment help you judge a resident's readiness for a clinical task?
- EPA and milestone assessment, how do they help you make supervision decisions?

**Appendix 2. Medical residents’ interview guide questions used to generate Q statements**

1. What do you think of the assessment tools currently used in emergency departments?

- What do you think of their quality?
- What do you think are the obstacles to the assessment, that is, what obstacles do you face?
- What improvements would you suggest?

2. Is feedback important to you? How important is it?

3. What do you think at the moment about the system of giving feedback (online or offline)? What are the advantages? With shortcomings?

4. What stands out as a negative experience in your history as a resident? Why did this experience count as negative? What stands out as positive feedback in your history as a resident? Why did this experience count as positive?

- How did the feedback obtained from this experience enhance your learning experience?

5. Is there a method of feedback that you particularly like or prefer?

- What kind of feedback, words vs scores do you think is helpful for improving your learning? Why?
- Ideally, how would you like to receive your feedback?

6. Can you give examples of barriers to receiving feedback?  What are your suggestions for improvement?

7. What kind of feedback do you think is credible? What kind of feedback can you trust?

- When your colleagues give you feedback, what kind of feedback makes you think, alas, I can believe this, this is trustworthy -What is the content of that feedback?
- What kind of feedback do you feel that is helpful and acceptable?
- What kind of factors make you feel like a clinical educators’ feedback is worth reflecting on?
- What factors increase your acceptance of feedback provided by the clinical teacher?
- How can clinical educators improve your acceptance of feedback?

8. What characteristics should a clinical teacher have for you to take their feedback as credible?

- Do you think the attitude of the teacher will affect your acceptance of feedback?

9. Do you perceive the amount of supervision or level of autonomy you have to practice independently as a form of feedback? If so, how?

10. Do you think the type of assessment tool being used to assess you and provide the basis of feedback is important to how you perceive feedback? How does the assessment tool affect your perception of feedback?

- If a clinical educator uses an assessment tool to evaluate you and then gives feedback based on that tool, how would that affect your level of feedback receptivity?
